# Acclimation of bacterial cell state for high-throughput enzyme engineering using a DmpR-dependent transcriptional activation system

**DOI:** 10.1038/s41598-020-62892-1

**Published:** 2020-04-08

**Authors:** Kil Koang Kwon, Soo-Jin Yeom, Su-Lim Choi, Eugene Rha, Hyewon Lee, Haseong Kim, Dae-Hee Lee, Seung-Goo Lee

**Affiliations:** 1 Synthetic Biology and Bioengineering Research Center, Korea Research Institute of Bioscience and Biotechnology (KRIBB), Daejeon, 34141 Republic of Korea; 2 School of Biological Sciences and Technology, Chonnam National University, Gwangju, 61186 Republic of Korea; 3 Department of Biosystems and Bioengineering, KRIBB School of Biotechnology, University of Science and Technology, Daejeon, 34113 Republic of Korea

**Keywords:** Synthetic biology, Standardization, High-throughput screening

## Abstract

Genetic circuit-based biosensors have emerged as an effective analytical tool in synthetic biology; these biosensors can be applied to high-throughput screening of new biocatalysts and metabolic pathways. Sigma 54 (σ^54^)-dependent transcription factor (TF) can be a valuable component of these biosensors owing to its intrinsic silent property compared to most of the housekeeping sigma 70 (σ^70^) TFs. Here, we show that these unique characteristics of σ^54^-dependent TFs can be used to control the host cell state to be more appropriate for high-throughput screening. The acclimation of cell state was achieved by using guanosine (penta)tetraphosphate ((p)ppGpp)-related genes (*relA*, *spoT*) and nutrient conditions, to link the σ^54^ TF-based reporter expression with the target enzyme activity. By controlling stringent programmed responses and optimizing assay conditions, catalytically improved tyrosine phenol lyase (TPL) enzymes were successfully obtained using a σ^54^-dependent DmpR as the TF component, demonstrating the practical feasibility of this biosensor. This combinatorial strategy of biosensors using σ factor-dependent TFs will allow for more effective high-throughput enzyme engineering with broad applicability.

## Introduction

Establishing a clear link between the genotype and phenotype is one of the fundamental considerations when developing biological sensors for screening^1,2^. Thus, the detectable phenomenon of screening tools should be directly correlated with the biological activity of the coding genes of interest. In this regard, transcription factors (TFs) are one of the best options for the rapid sensing and visualization of product molecules generated from target enzyme reactions by triggering reporter gene expression^3–5^. TFs in cellular networks naturally respond to specific intracellular or extracellular molecules, followed by feedforward or feedback control for transcriptional regulation. In bacteria, sigma factor (σ) is an initial TF protein that binds the promoter DNA region, associates with core RNA polymerase (RNAP; subunits: α_2_, β, β′ and ω) and melts the DNA double strand for transcription initiation^6–9^. Among all TFs, sigma 54 (σ^54^, *rpoN*)-dependent TF would be a promising sensory protein in the development of biosensors given its well-documented response to specific molecules under starvation conditions^10,11^. This unique property of σ^54^-dependent TFs has attracted interest with regards to the design of a genetic circuit to detect specific condition-dependent molecules^5,12^. Unlike the housekeeping TF σ^70^ (*rpoD*) and other σs (σ^19^-*fecI*, σ^24^-*rpoE*, σ^28^-*rpoF*, σ^32^-*rpoH* and σ^38^-*rpoS*), σ^54^ requires bacterial enhancer binding proteins (bEBPs) to distort the DNA double strand, and this could affect ligand-dependent transcriptional activation/repression, thereby serving as useful biosensing tools.

From a mechanistic point of view, σ^54^-dependent TFs are activated when guanosine tetraphosphate (ppGpp) and guanosine pentaphosphate (pppGpp) levels are elevated in cells under stress conditions^13,14^. (p)ppGpp concentrations can be controlled by RelA (GTP pyrophosphokinase)/SpoT (ppGpp synthase) homologs (RSH) as a stringent response to environmental stress (e.g., carbon/nitrogen limitation). When RelA binds to the 70 S ribosome, (p)ppGpp is synthesized in the state of amino acid deprivation^13,15^. As a (p)ppGpp synthase, SpoT has dual functions of hydrolysis and synthesis of (p)ppGpp, and these functions are triggered by various environment signals, whereas RelA only possesses synthesizing activity owing to its inactive hydrolysis domain. The generated (p)ppGpp then binds to RNAP with the RNAP-binding TF DksA, which switches on the upregulation of several TFs/translation factors, including σ^54^-dependent TFs^16,17^. Typical σ^54-^dependent TFs include phenol-responsive DmpR^18^, toluene-responsive XylR^5^, isoprene-responsive TbuT^19^, benzyl alcohol-responsive AreR^20^, 2-hydroxybiphenyl-responsive HbpR^21^ and nitrogen oxide-responsive NorR^22^.

**Figure 1:**
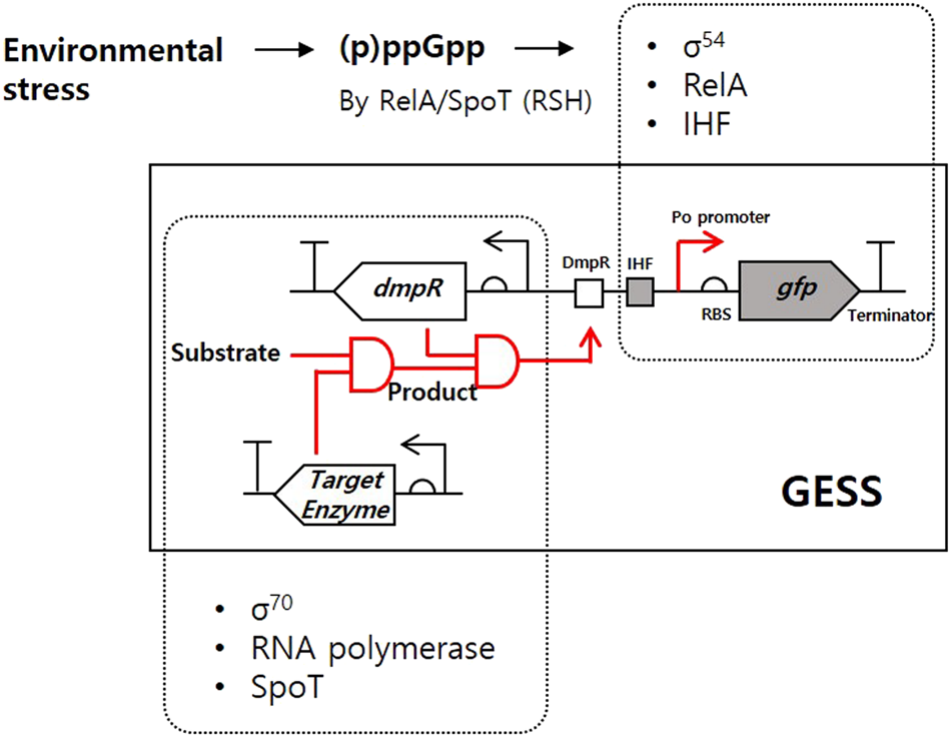
Schematic depiction of the GESS and involved transcriptional factors in the host. Under stress conditions, (p)ppGpp can be synthesized by RelA/SpoT, which controls the expression levels of several transcription factors. Along with the target enzyme, the GESS consists of sigma 70 (σ^70^, *rpoD*)-dependent DmpR/enzyme expression systems and a σ^54^-dependent reporter expression system, including RNA polymerase (subunit α, β, β′ and ω corresponds to *rpoA, rpoB, rpoC* and *rpoZ*, respectively), SpoT (bifunctional (p)ppGpp synthase/hydrolase), RelA ((p)ppGpp synthase), σ^54^ (sigma 54 factor, *rpoN*) and IHF (heterodimer subunits, *ihfA* and *ihfB*).

We previously reported a genetic enzyme screening system (GESS) wherein the σ^54^-dependent TF DmpR has been used to sense phenolic compounds in *Escherichia coli*^23,24^. Other than DmpR, GESS consists of the Po promoter of the *dmp* operon from *Pseudomonas* sp. and reporter proteins to facilitate analysis of enzyme-derived catalytic activity^23,24^. Thus, DmpR-GESS is a TF-based biosensor that senses phenolic compounds as products of enzymatic reactions. DmpR-GESS comprises two AND logic gates—the first consists of substrates and the target enzyme, which relies on the protein expression system of the host (Fig. 1), the product generated from whom becomes the input signal of the second AND gate along with the DmpR-based reporter system.

In DmpR-GESS, σ^54^ binds to the Po promoter sequence (−24, −12) of the green fluorescent protein (GFP) reporter gene with RNAP, whereas the σ^54^-RNAP complex is constrained at transcriptional initiation until the phenol-DmpR oligomer binds to its upstream binding region. Via an enzymatic reaction, the target enzyme generates phenol, which binds to DmpR and triggers σ^54^-dependent transcriptional initiation with the integration host factor (IHF), thereby allowing observation of the fluorescent signal representing enzymatic activity (Fig. 1).

Under normal growth conditions, σ^70^, as another sigma factor, binds the promoter sequences (−35, −10) of protein-coding genes and recruits RNAP for target gene transcription. When growth phase of GESS cells transit from the exponential to the stationary stage, environmental stress-induced (p)ppGpp is synthesized by RelA/SpoT to repress ribosomal RNA production, thereby decreasing target enzyme expression^13^. Meanwhile, (p)ppGpp also controls several genes involved in general metabolism along with global regulators, including σ^54^.

Based on this naturally programmed sigma factor mechanism, we hypothesized that the efficiency of GESS could be improved by sequential activation of the σ^70^-dependent target enzyme followed by σ^54^-dependent sensing of phenol molecules. In other words, the activity of the σ^54^-dependent DmpR gene was needed to be supressed by supplying a rich medium that would retain σ^70^-related target enzyme activity. After this enzyme reaction stage with minimum leakage of GFP reporter gene expression, replacing the medium with another medium having an appropriate carbon source would trigger a stringent response to rapidly maximize reporter gene expression.

Various substrates that may release phenol or *para*-nitrophenol could be used for different enzyme activities because these phenolic compounds generated from the enzyme reaction can induce intracellular fluorescent protein expression via phenolic compound-dependent transcriptional activation of DmpR. Indeed, we have reported the successful applications of GESS in enzyme assays and screening^24,25^. However, in enzymatic assays using DmpR-GESS, the effects of the cellular state was not studied in previous studies and culture conditions were tied strictly to the stringent conditions. Thus, it is important to optimize the assay condition of DmpR-GESS to improve the signal-to-noise ratio of genetic circuit-based screenings.

In this study, we investigated the characteristics of σ^54^-dependent biosensors and established an efficient high-throughput screening protocol through by optimising a specific host cell strain, nutrient-limiting conditions, enzyme reaction stages and biosensor detection in liquid and solid media. To apply our screening protocol using DmpR-GESS, we selected tyrosine phenol-lyase (TPL, EC 4.1.99.2) as the model enzyme and successfully identified a mutant TPL enzyme with improved catalytic activity using a random mutagenesis library.

## Results

### *E. coli*Δ*relA* Strains Showed Low Background Noise

**Figure 2:**
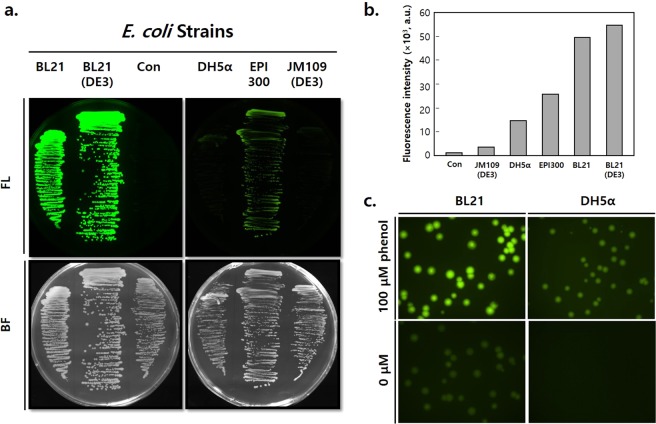
Fluorescence intensity of the GESS using various *E. coli* hosts. (**a**) Microscopic image of colonies harbouring the plasmid pGESS grown on LB agar plates containing 100 μM phenol; Con: control strain (DH5α) harbouring the pUC19 plasmid. Image processing and analysis were performed using Nikon’s NIS-Elements AR 4.2 software. (**b**) Fluorescence intensity of cells harbouring the pGESS grown on LB broth plates containing 100 μM phenol. (**c**) Microscopic image of colonies harbouring pGESS in BL21 and DH5α on LB agar plates.

To identify the optimal *E. coli* strain for DmpR–GESS reactions, we first compared biosensor responses to phenol in various *E. coli* strains (DH5α, EPI300, JM109(DE3), BL21 and BL21(DE3)) that harbour the plasmid pGESS (Fig. 2). BL21 and BL21(DE3) exhibited strong fluorescence on lysogeny broth (LB) plates containing 100 μM phenol, whereas DH5α harbouring the pUC19 plasmid (negative control) showed no fluorescence (Fig. 2a,b). Under the same experimental conditions, DH5α, EPI300 and JM109(DE3) as K-strains showed weaker fluorescence than the B-strains. There are many genetic differences between the B- and K-strains, and *relA* plays an important role in the discrepancy of phenol responses because it is involved in (p)ppGpp biosynthesis. Indeed, the K-strains used in this study had been genetically modified with a *relA* deletion for DNA cloning. To test the direct effect of *relA* in the host cells, recombinant RelA was expressed in JM109(DE3) cells harbouring pGESS. RelA-expressing JM109(DE3) showed stronger fluorescence than the RelA-deletion cells (Fig. S1), indicating that RelA could boost the σ^54^-dependent expression of the fluorescent protein via the DmpR–GESS genetic circuit. Such host dependency would be a selectable characteristic of DmpR–GESS, with combined advantages of a strong fluorescent response and low background noise. To further examine the host dependency of DmpR–GESS, we adopted the representative laboratory *E. coli* strains BL21 and DH5α, which are *relA*-positive and -negative, respectively, to detect fluorescent signals. BL21 showed stronger fluorescence than DH5α with the same concentration of phenol, whereas DH5α showed lower background noise than BL21 (Fig. 2c). The background signal of the BL21-GESS should be minimized for particular screenings that may lead to the risk of generating false positives. In this regard, here the DH5α was chosen as the host of the biosensor for subsequent tests to minimize the noise signal during the TPL screening steps.

### Stringent response-inducible medium is better for the sensing stage

**Figure 3:**
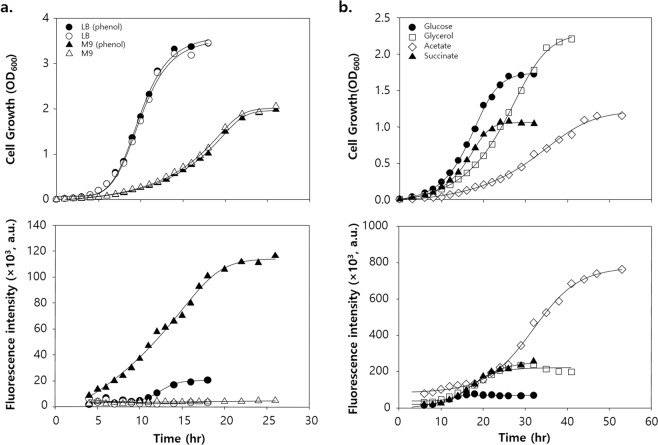
Time-lapse cell growth and fluorescence intensity profiles of GESS with various culture media. (**a**) Cell growth and corresponding fluorescence intensity profiles of DH5α harbouring the pGESS grown in LB and M9 4 g/L glucose broth; “phenol” indicates that the broth contained 100 μM phenol. (**b**) Cell growth and fluorescence profiles of DH5α harbouring pGESS grown in various carbon sources in M9 broth containing 100 μM phenol.

Compared with enriched medium, minimal medium would be unfavourable for *E. coli* cell growth and foreign protein expression. In contrast, DmpR-inducible Po promoter transcriptional activity can immediately occur in minimal medium, regardless of the growth stage^26,27^. Moreover, as a σ^54^-dependent TF, DmpR can be affected by the medium’s nutrient composition. Thus, to evaluate the correlation between transcriptional activity and nutrients in the medium, DH5α cells harbouring DmpR–GESS were grown in LB medium or M9 minimal medium supplemented with 4 g/L glucose; both media contained 100 μM phenol (Fig. 3). The growth of DH5α cells was much faster in LB than in M9 (Fig. 3a, upper panel), but the intracellular fluorescence in response to phenol by DmpR–GESS was more immediate and enhanced in M9 than in LB (Fig. 3a, lower panel). Moreover, fluorescent protein expression was first detected during exponential-to-stationary phase transition (after 10 h) in LB medium but was detected earlier in the M9 minimal medium. Stringent response in cells could be induced by depletion of the carbon source in the medium as well as under stress conditions due to lack of amino acids^28^. Thus, these results indicate that the response of DmpR–GESS may be controlled by stringent responsive regulatory factors such as (p)ppGpp and σ^54^. To further investigate the effect of different carbon sources on circuit performance, four different carbon sources (glycerol, glucose, sodium acetate and sodium succinate) were tested in M9 medium containing 100 μM phenol (Fig. 3b). Among the four carbon sources, acetate was the most unfavourable substrate for *E. coli* growth (Fig. 3b, upper panel), and it showed the highest fluorescence intensity among all media (Fig. 3b, lower panel). Therefore, M9 medium using acetate would help enhance the response of DmpR–GESS.

**Figure 4:**
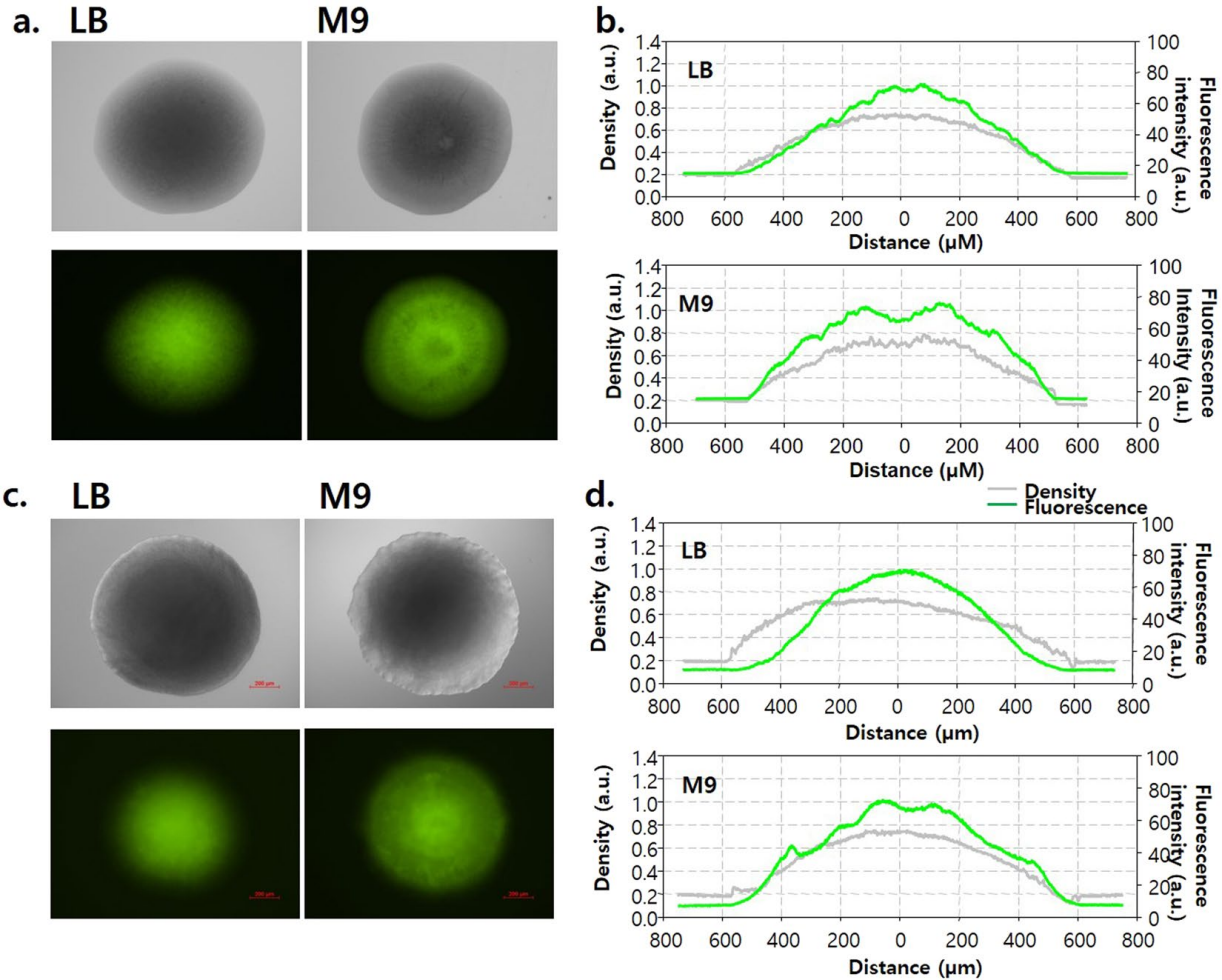
Fluorescence intensity of colonies harbouring pGESS or pHCEIIB-*egfp* on LB and M9 4 g/L glucose agar plates. (**a**) Microscopic image of single colonies harbouring the pHCEIIB-EGFP on LB and M9 agar plates. Image processing and analysis were performed using Nikon’s NIS-Elements AR 4.2 software. (**b**) Fluorescence intensity and colony density profile. (**c**) Microscopic image of single colonies harbouring pGESS on LB and M9 agar plates containing 100 μM phenol. (**d**) Fluorescence intensity and colony density profile.

To compare the response of DmpR–GESS toward phenol in a single-colony state, images of similar-sized colonies from the LB and M9 glucose agar plates were obtained under fluorescence and bright-field imaging conditions, and the fluorescence intensity profiles were analysed along horizontal edges (Fig. 4). DH5α harbouring pHCEIIB-*egfp* (control), in which the expression system is under control of the σ^70^-related promoter, showed a distributed fluorescence signal in a single-colony state, regardless of the medium composition (Fig. 4a,b). However, for DmpR–GESS with phenol, the fluorescence intensity at the edge of the colony was clearly lower on LB plate than on the M9 plate (Fig. 4c,d). Similar to the effects of rich broth medium on the response of DmpR-GESS to phenol, this effect may be derived from the poorer nutrient conditions at the colony centre, in opposite to the conditions at the colony edges. Also, on M9 succinate and M9 acetate agar plates, similar sizes and fluorescence profiles of colonies were observed after incubated longer for 72 and 120 h, respectively (Fig. S2). Thus, M9 glucose agar plate was chosen for the subsequent enzyme screening steps.

### Acclimated biosensor cells indicated tpl mutant enzyme with higher activity

**Figure 5:**
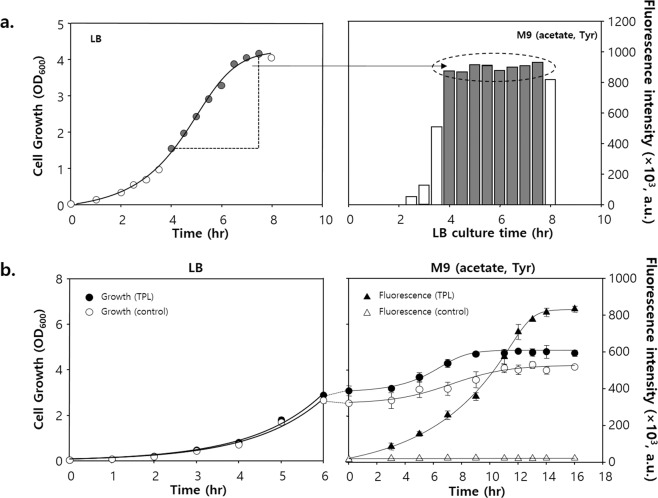
Cell growth and fluorescence intensity of GESS expressing TPL determined with a two-step reaction. (**a**) Fluorescence intensity of GESS in response to TPL activity at different cell states in LB broth. (**b**) Time-lapse cell growth and fluorescence intensity by the two-step reaction. The cells were cultured in LB broth until the late exponential growth phase as the first step, and the detection reaction was performed in M9 acetate medium with tyrosine as the second step. Circles represent the OD_600_ value, and triangles represent the specific fluorescence intensity (GFP/OD_600_).

To apply the established screening condition using DmpR–GESS, we chose TPL as the model enzyme. TPL is a pyridoxal-5′-phosphate-dependent enzyme that catalyses the β-elimination of tyrosine and produces ammonium pyruvate and phenol^29,30^. DH5α cells harbouring both TPL and DmpR–GESS were cultured in various media, including LB and 4 g/L glucose and 4 g/L acetate M9 media, containing 1 mM tyrosine as a substrate for the production of phenol via enzymatic reaction. As expected, the cells grown in LB showed rapid growth, whereas those grown in M9 medium exhibited growth-independent high fluorescence response (Fig. S3). If the σ^70^-related protein expression and σ^54^-dependent fluorescence response could be separated by simply changing the nutrient compositions in the media, the maximum fluorescence intensity corresponding to the enzymatic activity could be detected within a shorter cultivation time. Therefore, we compared fluorescence intensities based on changes from rich to minimal medium in terms of the two-step reaction. *E. coli* DH5α cells containing both TPL and DmpR–GESS were first grown in LB medium and then samples were collected at different growth phases (Fig. 5a). The medium was changed after mild centrifugation, and these samples were then incubated in M9 acetate medium with tyrosine (which exhibited the highest fluorescence response, as described above) for 16 h. As a result, the fluorescence intensity of TPL-expressing cells harvested when OD_600_ was between 1.5 and 4 in LB medium reached their saturation level after reaction in M9 acetate medium (Fig. 5a, right panel). Therefore, medium change was appropriate after 6 h in LB when the OD_600_ reached approximately 3 (Fig. 5b, left panel). Cells were grown in LB medium for 6 h, transferred to M9 acetate medium and monitored for fluorescence intensity (Fig. 5b, right panel). The fluorescence intensity of cells became stronger with increasing reaction time and saturated at 14–16 h, whereas cells without the TPL gene showed no fluorescent signal. This difference is associated with the stringent response under controlled environmental conditions. Thus, by distinguishing cell growth based on enzyme reaction stage, the response of the genetic circuit can be improved.

**Figure 6:**
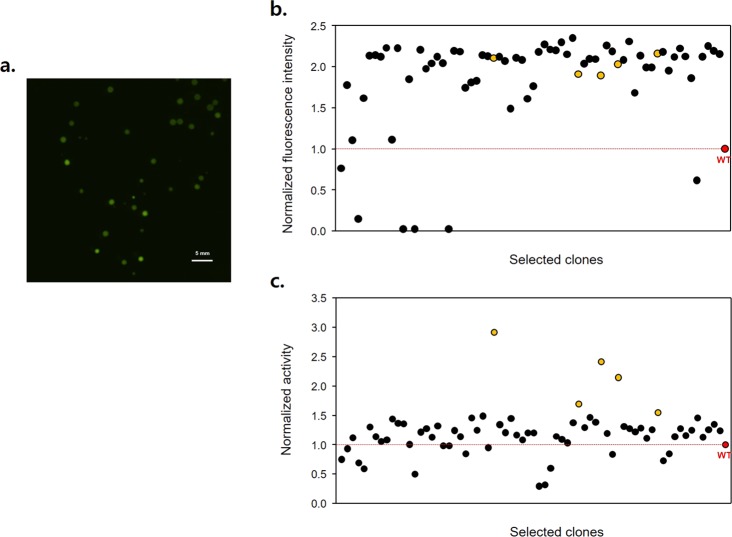
Application of GESS to screen mutant TTPL using M9 minimal medium plates and the two-step reaction. (**a**) Microscopic image of colonies harbouring pGESS and mutant TTPL. Image processing and analysis were performed using Nikon’s NIS-Elements AR 4.2 software. (**b**) Normalized fluorescence intensity of mutant TTPLs by the GESS two-step reaction for screening high activity. Final candidates are represented as yellow circles. (**c**) Normalized enzyme activity of mutant TTPLs by heat inactivation and 4-AAP measurement for screening high stability.

To test the feasibility of the practical applications of GESS in enzyme screening, thermostable TPL (TTPL) from *Symbiobacterium toebii* was used as the target enzyme^29^. Random mutagenesis was performed by error-prone polymerase-chain reaction and transformed into the biosensor *E. coli* cells. After electrophoresis, the cells were spread on M9 glucose agar plates containing tyrosine, and colonies exhibiting strong fluorescence intensity were selected for further screening (Fig. 6a). The colonies were grown for 6 hours in LB-microplates, transferred to incubate in M9-acetate media, and analysed for the cellular fluorescence to obtain high activity mutants (Fig. 6b). The mutants were tested then for thermal stability by heat treatment (Fig. 6c). The mutations of these candidates and kinetic parameters are listed in Table S1 and S2. Mutant #1 and #2 showed higher catalytic activity than wild type (2- and 5-fold) and #2 represented a 50% increase in terms of *k*_*cat*_/*K*_*M*_. Among these candidates, the mutation V193I from mutant #2 is located near the substrate-binding residues and the P16L from mutant #1 is located near the dimer–dimer interface, which might affect the catalytic activity and stability of the enzyme (Fig. S4).

## Discussion

Synthetic biosensors using TFs have been recognized as powerful tools for synthetic biology and enzyme engineering. Here, we characterized a genetic circuit using DmpR as a σ^54^-dependent TF with unique transcriptional activation and demonstrated its applications in enzyme screening. DmpR, a member of the nitrogen regulatory protein C (NtrC) family, comprises a signal reception domain (A domain), an AAA + family ATPase domain (C domain), a DNA-binding domain (D domain) and a hinge domain connecting the A domain and C domain (B domain)^18,31^. Similar to the NtrC family proteins, DmpR might exist as an inactive dimer that represses the function of ATPase in the absence of a ligand^32,33^. After the ligand binds the A domain, ATP hydrolysis by the released C domain triggers DmpR oligomerization and binding to promoter upstream regions. The conserved loop of ATPase interacts with the −12 DNA region of the promoter with DNA bending by the IHF and helps melt the DNA double strand to initiate transcription^8,9^. Along with DmpR as the bEBP, σ^54^ is another important factor affecting the transcriptional initiation of the reporter gene in the DmpR–GESS. IHF, DksA and (p)ppGpp are essential factors for activation of the Po promoter *in vivo*; however, the IHF, which can induce DNA bending, can only affect transcriptional initiation *in vitro*^34,35^. Because IHF expression is dependent on the level of (p)ppGpp^36^, the σ^54^-dependent promoter would be regulated by synthesis of (p)ppGpp in a stringent response.

RelA and SpoT play key roles in the biosynthesis of (p)ppGpp in *E. coli*^37^. Here, we demonstrated strong fluorescence intensity by the presence of *relA* gene. In the presence of phenol, strong fluorescence intensity of the GESS indicates a high level of (p)ppGpp by RelA expression, whereas a weak fluorescence intensity indicates a low level of (p)ppGpp in the *relA*-null host. Therefore, it is presumed that we can selectively apply these cell states based on sensitivity demands. RelA-expressing *E. coli* can be used for determining phenol concentrations in the screening requiring high levels of sensitivity, whereas SpoT-only-expressing hosts, such as DH5α in this study, can be used for enzyme screening with low false positives.

SpoT also plays a role in the response to various types of environmental stresses, such as carbon, iron or fatty acid depletions^37–40^. Although the detailed molecular mechanism of regulation by SpoT is yet unclear, holo-acyl carrier protein (ACP, carrying 4′-PP) and regulator of sigma D (Rsd) can bind to SpoT to inhibit (p)ppGpp hydrolysis while promoting (p)ppGpp synthesis^38,39^. Further, the binding of SpoT with ACP and Rsd was induced by amino acid deficiency or unfavourable carbon sources^38,39^. Therefore, the different fluorescent signals observed in minimal media with different carbon sources in the *relA*-null host may be due to the different levels of SpoT activity. Consequently, in the σ^54^-dependent TF-based biosensor, which is affected by stringent response, the output fluorescent signal could be improved by using an unfavourable carbon source, which was determined to be acetate in the present study.

As cells cultured on agar plates form colonies, aging cells accumulate in the centre of the colony, whereas newly dividing cells grow along the edges. Indeed, we observed clear differences in the growth stages of cells within a single colony. On the LB plates, GESS reflected these different growth stages by the centre-condensed fluorescence, whereas growth in M9 minimal solid medium provided an even fluorescence distribution in a single colony that would be useful for obtaining a stable output signal by a phenolic compound.

In the cell assay of enzyme activity using GESS, recombinant proteins are normally expressed under σ^70^-related promoters owing to the high expression levels in rich media (Fig. S5). Likewise, the expression of fluorescent proteins in the genetic circuit, which depends on the σ^54^ promoter, would be silenced for the reduction of noise. Changing the medium from LB to M9 minimal medium in the late-exponential phase as a two-step culture process was able to satisfy the conditions necessary for maximum fluorescence response as well as the high expression level of the target enzyme. Finally, we applied this optimised DmpR–GESS condition for screening TTPL, the industrial applications of which have not been focussed on owing to its unsatisfactory catalytic activity. With this system, the fluorescence signal-based catalytically improved mutants were successfully screened under optimised screening methods. The P16L mutants of TTPL are located near the oligomeric interface, an important site for an enzyme’s catalytic activity and stability^30^. This result indicates that screening using a biosensor under the proposed optimised conditions could offer a useful tool for rapid and convenient enzyme engineering.

## Conclusion

A TF-based biosensor combined with a σ^54^-dependent promoter could provide various applications, including enzyme screening. To optimize the reactivity and stability of the genetic circuit, we attempted various methods to control stringent responses in host cells, such as host dependency, minimal media containing different carbon sources, physical conditions as liquid and solid phases and separation of growth and reaction stages as a two-step reaction. Based on this optimisation and the use of simple screening steps, mutant enzymes with improved catalytic activity were screened. This simple and rapid profiling may contribute to facilitating the functional analysis of enzymatic reactions and further synthetic biological engineering of new enzyme functions.

## Methods

### Materials

All chemicals were purchased from Sigma-Aldrich (St. Louis, MO, USA). DNA polymerase, restriction endonucleases and T4 DNA ligase were purchased from New England Biolabs (Beverly, MA, USA). All oligonucleotides used in this study were synthesized by Macrogen (Daejeon, Korea). DNA isolation and related techniques were performed according to the standard protocols for molecular biology^41^. Plasmid DNA isolation and DNA extraction from agarose gels were performed using Qiagen kits (Qiagen, CA, USA).

### Strains and plasmids

The plasmid pGESS constructed in our previous study was used as the genetic circuit system; it comprised a *dmpR* transcriptional activator and Po promoter from *Pseudomonas putida* KCTC 1452 as well as GFP as the reporter^23^. The pGESS was transformed into five *E. coli* strains [BL21, BL21(DE3), DH5α, EPI300 and JM109(DE3)] obtained from Novagen, Invitrogen, Epicentre and Promega using the calcium chloride transformation method to compare plasmid expression in different strains^41^.

The relA gene from *E. coli* MG1655 was cloned into a modified pET21a plasmid using NdeI and HindIII restriction enzymes, in which the origin and antibiotic resistance gene were replaced with those of pACYC (forward primer: 5′-GAT ATA CAT ATG GTT GCG GTA AGA AGT GC-3′; reverse primer: 5′-GGC CGC AAG CTT CTA ACT CCC GTG CAA CCG ACG-3′). The constructed plasmid was then transformed into JM109(DE3) cells harbouring pGESS for analysing the influence of RelA. Thermostable TPL (TTPL) from *S. toebii* was cloned into the pSHCE plasmid using NdeI and HindIII and transformed into DH5α cells harbouring pGESS (forward primer: 5′- GAT ATC ATA TGC AGC GAC CCT-3′; reverse primer: 5′- ACA GCC AAG CTT AGC TGA TCG GCT CGA AG-3′)^24^.

### Fluorescence analysis

Cells were grown on LB medium (10 g tryptone, 5 g yeast extract and 5 g NaCl per litre) and M9 minimal medium (12.8 g Na_2_HPO_4_·7H_2_O, 3 g KH_2_PO_4_, 0.5 g NaCl, 1 g NH_4_Cl, 2 mM MgSO_4_, 0.1 mM CaCl_2_, 0.4% (w/v) carbon sources and 0.01% (w/v) thiamine per later) supplemented with 50 μg/mL ampicillin and 100 μM of phenol as the effector of the *dmpR* regulator at 30 °C for 48 h. Image analysis was performed on a fluorescence microscope (AZ100M, Nikon, Japan) combined with epifluorescence and diascopic DIC accessories. Images were acquired with a monochrome CCD camera (DS-Qi1Mc, Nikon, Japan) using a fluorescence filter set (GFP-HQ, Nikon, Japan) (Ex 455–485 nm, DM 495, BA 500–545). Image processing and analysis were performed using Nikon’s NIS-Elements AR 4.2 software (https://www.microscope.healthcare.nikon.com/products/software/nis-elements).

For liquid cultivation, 1% seed culture was inoculated and cultured at 30 °C in LB or M9 containing 100 mM phenol and 50 mg/ml ampicillin. For the two-step reaction, the cells were cultured in LB broth at 37 °C until reaching OD_600_ 1.5–3.0. The cells were harvested by centrifugation (1000 × *g*, 5 min), resuspended and cultivated in M9 medium with 4 g/L acetate at 30 °C. The fluorescence intensity in the broth was analysed using a multi-label reader (Perkin-Elmer, USA), and cell growth was measured on a UV-visible spectrophotometer (Pharmacia Biotech, USA). Fluorescence analysis with RelA was performed as described above with 0.1 mM isopropyl β-d-1-thiogalactopyranoside and appropriate antibiotics.

### TTPL mutant library screening

The random mutant library was constructed using the Genemorph II Random Mutagenesis kit (Agilent, USA), following the manufacturer’s protocol (0–4.5 mutations/kb). The random mutant library was transformed into the DH5α cells harbouring pGESS and spread on an M9 agar plate with 4 g/L glucose, 50 μM pyridoxal-5′-phosphate (PLP) and 1 mM tyrosine. Colony fluorescence was analysed by fluorescence microscopy after 48 h of culture at 30 °C. Cells emitting strong fluorescence were selected for the subsequent in-cell assay using the two-step reaction. To test the thermostability of the mutants, cultured cells were harvested and treated with Cellytic B at 37 °C for 30 min. After incubation at 65 °C for 30 min for heatshock, the cell lysate (100 μL) was mixed with an equal volume of substrate solution (potassium phosphate buffer containing 50 μM PLP and 1 mM tyrosine; pH 8.0). After enzyme reaction at 50 °C for 30 min, the reaction solutions were heated for 3 min at 94 °C, centrifuged to remove any insoluble aggregates and analysed for phenol production using colorimetric detection. In brief, 100 mL of the enzyme assay solution was added to a mixture of 100 μL of 0.1 M NaOH, 30 μL of 0.6% (w/v) 4-aminoantipyrine and 30 μL of 0.6% (w/v) potassium persulfate and incubated for 10 min to measure the absorbance on a multi-label reader. To determine the kinetic parameters of TTPL mutants, cells were cultured, harvested, disrupted by sonication and centrifuged to remove cell debris. The supernatant was used directly to purify the His-tagged enzymes by loading onto a Profinia Affinity Chromatography Protein Purification System (Bio-Rad, Hercules, CA, USA). Purified TTPL activity was monitored for 5 min in 50 mM potassium phosphate buffer containing 50 µM PLP and various concentrations of l-tyrosine. The concentration of the generated phenol was analysed using an HPLC system (Agilent Technologies, Santa Clara, CA, USA, UV 270 nm) using an instrument fitted with an Eclipse XDB-C18 column (4.6 × 150 mm^2^), with the mobile phase consisting of 50% acetonitrile and 50% water at a flow rate of 1 mL/min.

### Homology modelling

Homology models of TTPL were produced using the Modeller software (version 9.13, https://salilab.org/modeller) using the crystal structure of *Citrobacter freundii* TPL (PDB: 2TPL) as the template^42^. The generated structures were energy-minimized using Modeller, and the best conformers were chosen for further analyses. The tyrosine as ligand was docked onto the resulting structures using an Autodock vina ligand-docking unit (version 1.1.2, http://vina.scripps.edu)^43^.

## Supporting Information

Supporting Information.
