# Supplementary material for: Acclimation of bacterial cell state for high-throughput enzyme engineering using a DmpR-dependent transcriptional activation system
Source: Sci Rep. 2020 Apr 8;10:6091. doi: 10.1038/s41598-020-62892-1 (PMC7142073; doi:10.1038/s41598-020-62892-1)
Supplement: Supplementary file 1 — Supporting Information. [file 41598_2020_62892_MOESM1_ESM.docx]

*Supplementary Information*

**Acclimation of bacterial cell state for high-throughput enzyme engineering**

Kil Koang Kwon^1^†, Soo-Jin Yeom^1, 2^ †, Su-Lim Choi^1^, Eugene Rha^1^, Hyewon Lee^1^, Haseong Kim^1,3^, Dae-Hee Lee^1,3^ and Seung-Goo Lee^1,3^*

**Figure S1.** Time-lapse cell growth and fluorescence intensity of RelA expressing JM109(DE3) harboring pGESS in LB broth containing 100 μM phenol.


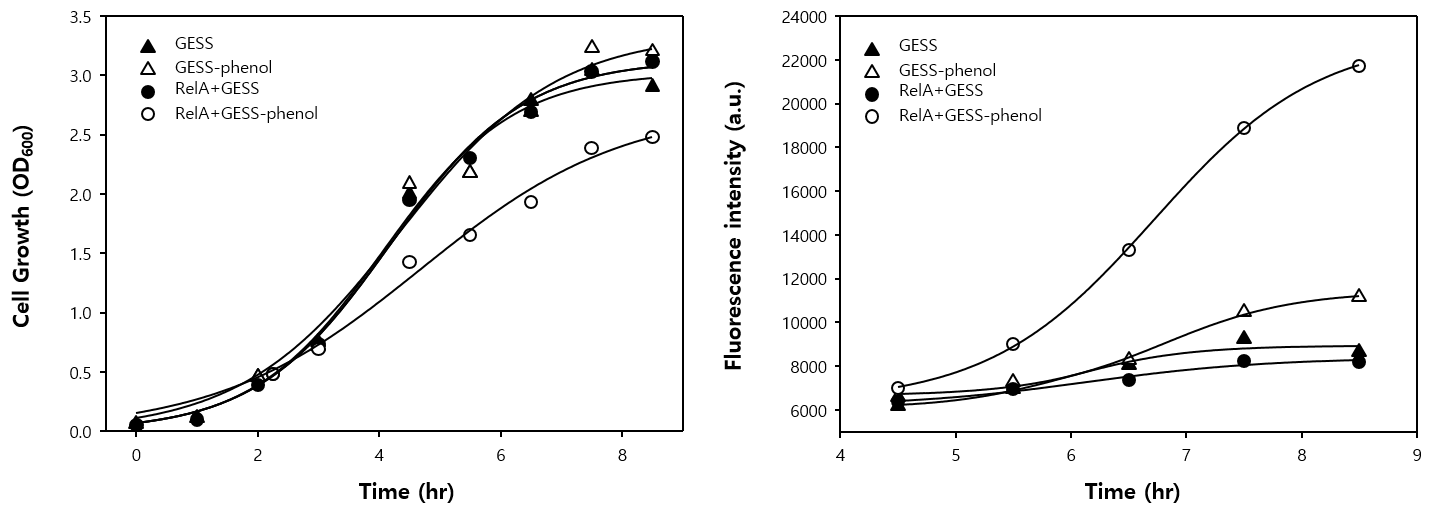


**Figure S2.** Fluorescence intensity and density of colonies harboring pGESS on M9 4 g/L succinate and acetate agar plates containing 100 μM phenol. Image processing and analysis were performed using Nikon's NIS-Elements AR 4.2 software.


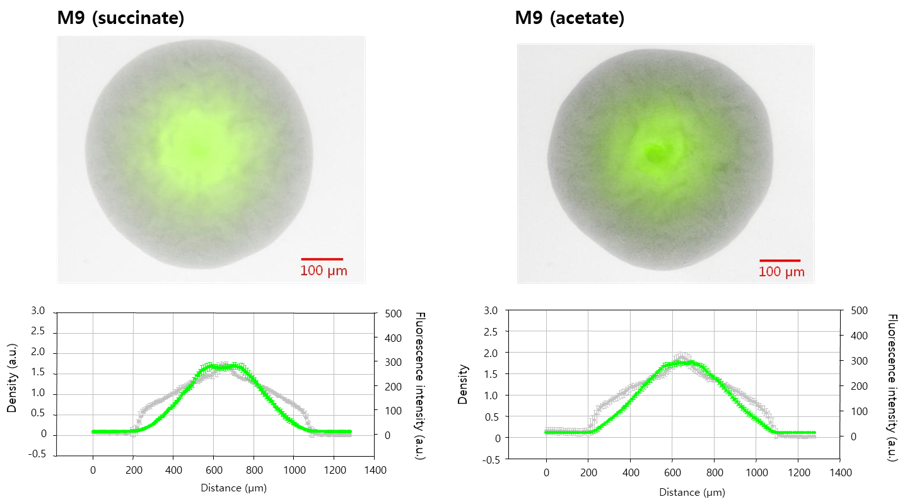


**Figure S3.** Cell growth and fluorescence intensity of GESS expressing TPL. Cell was cultured in LB, M9 4 g/L glucose and 4 g/L acetate containing 1 mM tyrosine at 30 °C.


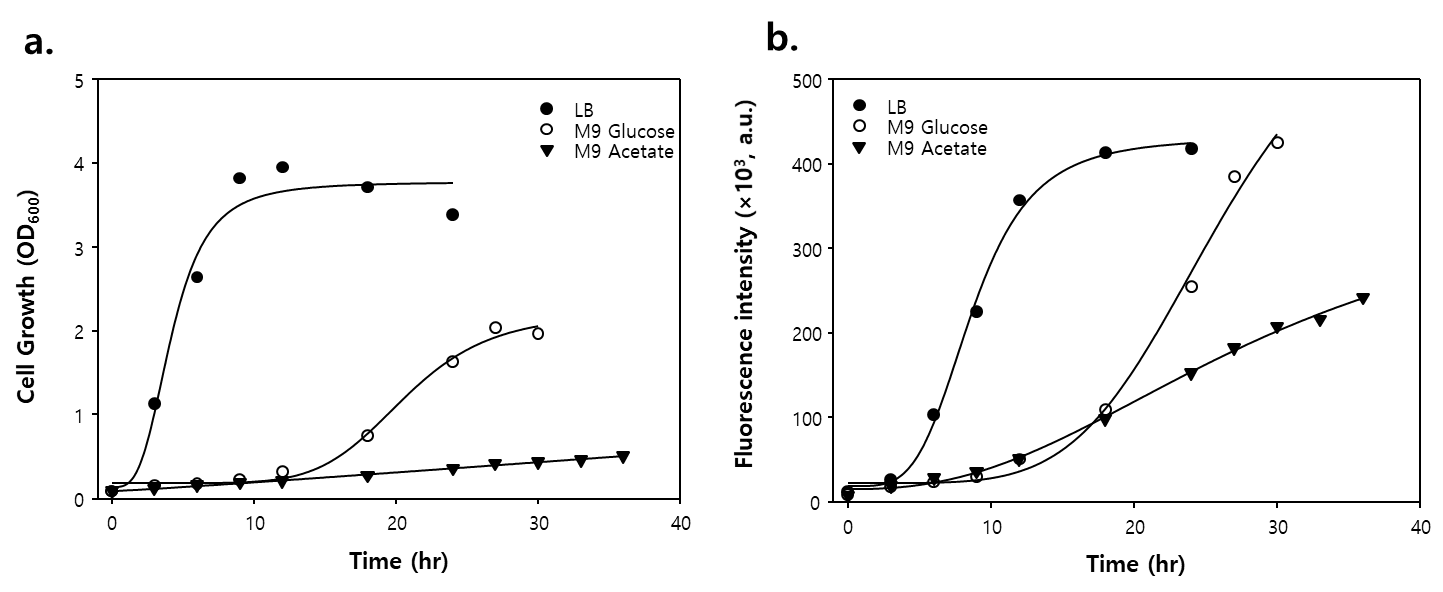


**Figure S4.** TTPL model structure. Tetrameric structure, tyrosine as a substrte and mutations (P16L and V193I) are represented as ribbon, ball-stick and yellow CPK styles, respectively. Homology models of TTPL and ligand docking were produced using the Modeller software (version 9.13) and Autodock vina (version 1.1.2), respectively.


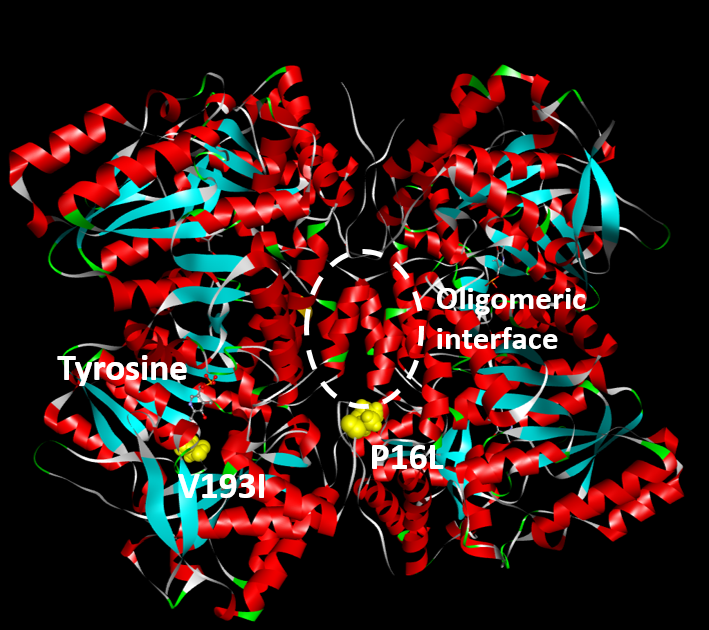


**Figure S5.** SDS-PAGE. Lane 1: Ladder. Line 2: *E. coli* cell extracts expressing TPL in M9. Line 3: *E. coli* cell extracts expressing TPL in LB.


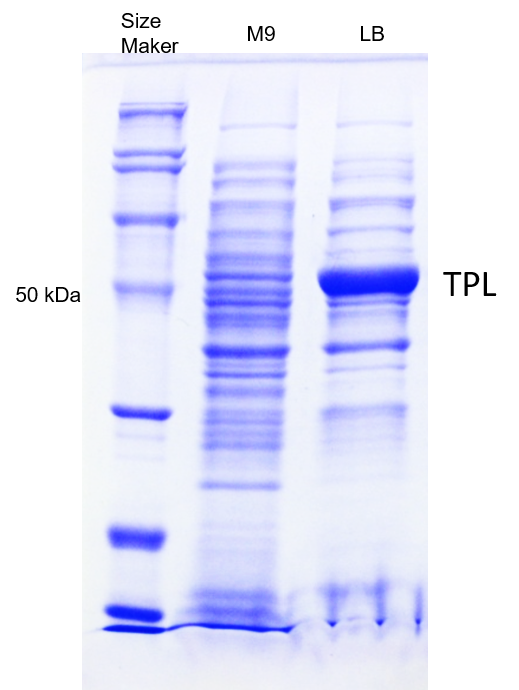


**Table S1**. Mutation residues of mTPLs from GESS screening

|  | **Mutations** |
| --- | --- |
| #1 | P16L, E165K |
| #2 | V193I, A350T, P444T |
| #3 | L111I, A302T, D312G |
| #4 | Y248C |
| #5 | M245V, Q359L, G388D |

**Table S2.** Kinetic parameters of thermostable TPL wild-type and mutants

|  | **WT** | **#1** | **#2** | **#3** | **#4** | **#5** |
| --- | --- | --- | --- | --- | --- | --- |
| *K_M_* (mM) | 0.27±0.01 | 0.49±0.08 | 1.07±0.10 | 0.34±0.02 | 0.29±0.07 | 0.46±0.10 |
| *k_cat_* (s^-1^) | 0.15±0.01 | 0.29±0.04 | 0.83±0.07 | 0.16±0.01 | 0.17±0.03 | 0.20±0.01 |
| *k_cat_/K_M_*  (s^-1^·mM^-1^) | 0.55±0.01 | 0.58±0.13 | 0.77±0.10 | 0.48±0.03 | 0.57±0.17 | 0.44±0.09 |

Purified thermostable TPLs were assayed at 60 °C in 5 mins. The reaction mixture (0.1 mL) contained 50 mM potassium phosphate buffer (pH 8.0), 0.05 mM PLP and various concentrations of tyrosine.
